# Effects of traditional agroecosystems and grazing areas on amphibian diversity in a region of central Mexico

**DOI:** 10.7717/peerj.6390

**Published:** 2019-02-15

**Authors:** José Daniel Lara-Tufiño, Luis M. Badillo-Saldaña, Raquel Hernández-Austria, Aurelio Ramírez-Bautista

**Affiliations:** Laboratorio de Ecología de Poblaciones, Centro de Investigaciones Biológicas, Instituto de Ciencias Básicas e Ingeniería, Universidad Autónoma del Estado de Hidalgo, Mineral de la Reforma, Hidalgo, México

**Keywords:** Alpha and beta diversity, Community, Conservation, Land-use change, Amphibians, Biodiversity, Mexico, Sierra Madre Oriental, Gulf of Mexico, Evenness

## Abstract

Habitat loss or degradation due to land cover change is regarded as one of the main drivers of amphibian decline; therefore, it is imperative to assess the effects of land-cover change on this group of vertebrates. In this study, we analyze changes in alpha and beta diversity of amphibian communities found in five land-cover types: mountain cloud forest, tropical evergreen forest, shade coffee, milpa huasteca, and grazing areas; six samples sites were established for each land-cover type, separated at least one km away. The study was conducted in the northwest part of the state of Hidalgo, in a transition zone between the Sierra Madre Oriental and the Gulf of Mexico, which is a region rich in amphibian species. The results indicate that alpha diversity decreases with loss of canopy cover, this being high in mountain cloud forest, tropical evergreen forest, and Shade coffee, and low in milpa huasteca and grazing areas. The land-cover type with the highest species evenness was found in milpa huasteca and the lowest in. The highest beta diversity was observed among tropical evergreen forest and grazing areas. Mountain cloud forest contains both exclusive species and the highest number of species currently regarded as threatened by national and international conservation assessment systems. In order to preserve amphibian diversity in the study area it is vital to protect the last remnants of native vegetation, especially mountain cloud forest, but also including Shade coffee, since the latter habitat harbors amphibian diversity similar to that found in native forests. Finally, implementation of policies that both reduce Grazing areas and increase their productivity is also necessary, since these highly modified areas turn out to be the ones that affect amphibian diversity the most.

## Introduction

Amphibians are considered the most threatened vertebrate group on the planet (Wilson, Johnson & Mata-Silva, 2013). It has been determined that during the last two decades, at least 34 amphibian species have gone extinct and 42% of existing species are threatened (IUCN, 2018; AmphibiaWeb, 2019). The main biotic and abiotic factors involved in their decline are habitat loss or degradation due to land use-change, infectious diseases, global warming, and introduction of exotic species (Green, 2003; Collins & Storfer, 2003). The effects of these factors are harmful for amphibians because they have low vagility, high vulnerability to pathogens, and low bioclimatic tolerance (Cushman, 2006; Bitar et al., 2012). Habitat loss or degradation due to land use change is regarded as one of the factors that threatens amphibians the most; therefore, understanding relationships between amphibians and their environment has become increasingly important for conservation of this vertebrate group (Weyrauch & Grubb, 2004; Collins & Crump, 2009). However, studies focused on the consequences of land use change on the diversity of amphibian communities have shown contrasting results to date, influenced primarily by specific bioclimatic characteristics of the study sites, the level of analysis (landscape), and by the species assemblages present at those sites (Urbina-Cardona, Olivares-Pérez & Reynoso, 2006; Trimble & Van Aarde, 2014).

It has been documented that some land use changes increase or sustain environmental heterogeneity, such as microhabitat types, which in turn increase the number of species and individuals (Henderson & Powell, 2001; Macip-Ríos & Casas-Andreu, 2008). This pattern has been found in traditional agricultural systems, such as shade coffee (SC) plantations, which show a biodiversity similar to that of primary forests, including birds, mammals, reptiles, insects, and plants (Mayfield & Daily, 2005; Macip-Ríos & Casas-Andreu, 2008; Williams-Guillén & Perfecto, 2010; Cruz-Elizalde et al., 2016). The retention of biodiversity by coffee plantations is likely explained by this habitat still containing the biotic and abiotic elements of the original undisturbed habitats (i.e., plant litter layers, soil, temperature, and moisture conditions). Additionally, the importance of these areas is that they enable species to establish themselves and also use these areas as bridges to move across areas with primary forest (Pimentel et al., 1992; López-Barrera, 2004). It has been documented that grazing areas (GA) and monocultures (i.e., cornfields, sun coffee plantations, and sugarcane fields) have, in contrast to SC plantations, negative effects on biodiversity by decreasing microhabitat complexity and moisture levels, increasing ambient temperatures and predation pressure, and ultimately reducing the number of species (Jansen & Healey, 2003; Santos-Barrera & Urbina-Cardona, 2011; Trimble & Van Aarde, 2014).

Habitat modification and fragmentation is clearly evident in neotropical countries, where a high diversity of amphibians and high numbers of threatened amphibian species are found (Stuart et al., 2010; Velázquez et al., 2002), and Mexico is an example of these countries. Currently, Mexico ranks as the fifth most diverse country with 378 amphibian species, of which more than 50% are country endemic (Wilson, Johnson & Mata-Silva, 2013). The highest species richness and endemism are found in cloud and tropical forest; however, unfortunately more than 70% of the original forest area in Mexico and Central America has been modified during the last 50 years (Velázquez et al., 2002; Stuart et al., 2010).

Modification of cloud and tropical forest in Mexico before 1970 was primarily the result of traditional agricultural systems such as milpa (an agroecosystem that mainly includes corn, beans and squash) and SC plantations. Then, at the beginning of the 1970s, traditional production systems were increasingly replaced by monocultures and GA, which as a consequence drastically modified environmental conditions (Velázquez et al., 2002; Challenger & Soberón, 2008) and likely threatened amphibian communities, as many species require specific environmental conditions (Cushman, 2006; Stuart et al., 2010). Considering that the land use change is a factor that is widely spread and diversified, the present work aims to evaluate the changes that occur in the alpha and beta diversity of amphibian communities when cloud forest and tropical forest are transformed into traditional agroecosystems and GA.

## Material and Methods

### Study area

**Figure 1:**
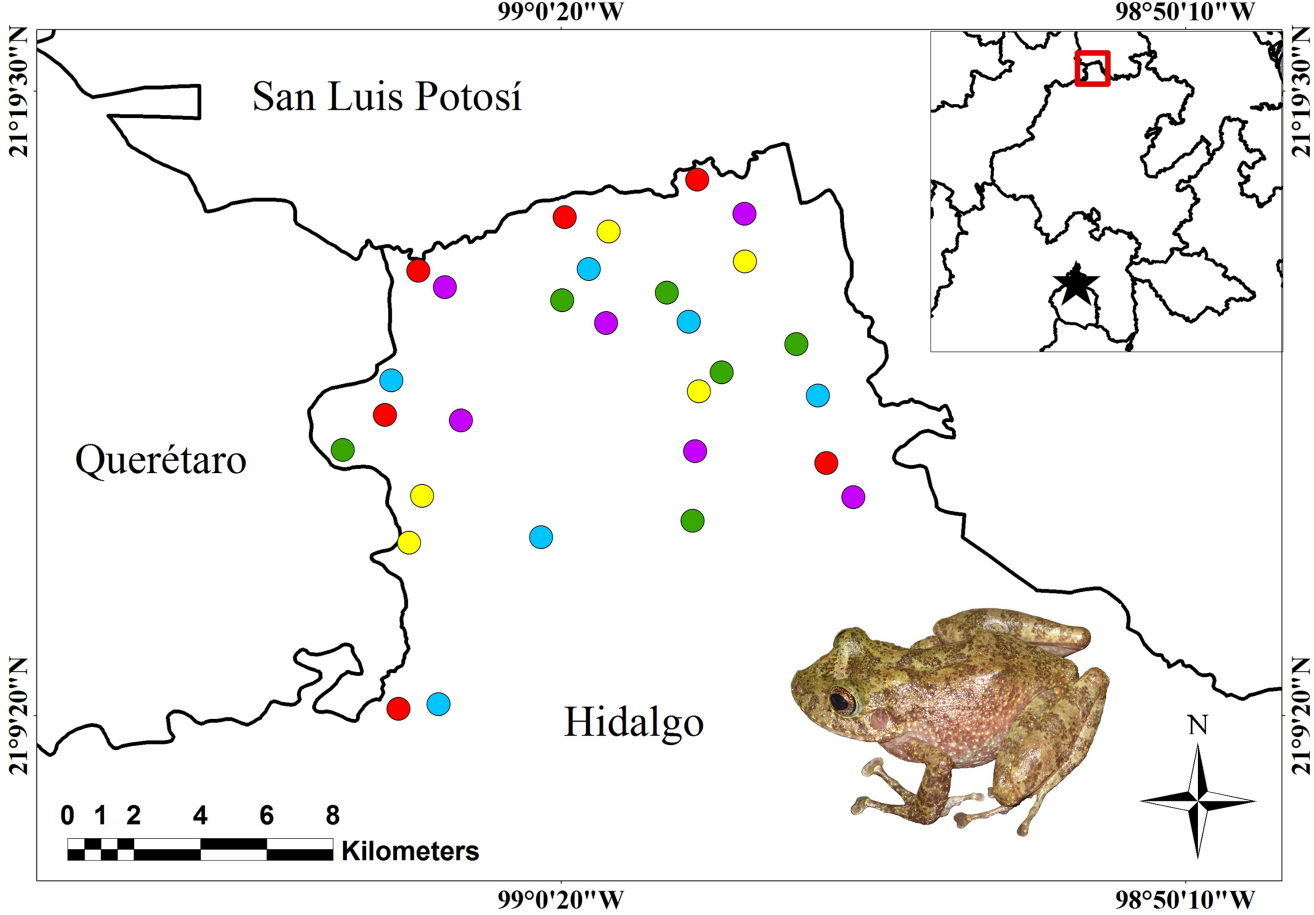
Location of the study area and sampling points. Study area. Sampling points are indicated for the land-cover types: green circles = mountain cloud forest; red circles = tropical evergreen forest; purple circles = shade coffee; yellow circles = milpa huasteca; blue circles = grazing area. Red square indicates the general location of the study area. Black star represents the location of Mexico City.

This study was carried out in the northwest of the state of Hidalgo (21°08′–21°09′ N; 98°53′–99°05′ W; Fig. 1) in a transition zone between the Sierra Madre Oriental and Gulf of Mexico. Currently, the area is represented by a series of vegetation remnants of what used to be undisturbed montane cloud forest (MCF) and tropical evergreen forest (TEF), immersed in a mosaic of agroecosystems, GA, and secondary vegetation (Fig. 1). The land-cover types analyzed were MCF, TEF, GA, SC, and milpa huasteca (MH). MCF is located at elevations between 900 and 1,500 m, on steep slopes and ravines (INEGI, 2009); with a mean annual temperature of 18 °C, and it receives 1,100–2,600 mm of precipitation annually (Rzedowski, 2006; INEGI, 2009). The dominant tree species belong to the genera *Alnus*, *Clethra*, *Liquidambar*, *Quercus*, and *Platanus*, with heights generally from 15 to 30 m. The site also harbors abundant epiphytes, such as lichens, mosses, bromeliads, and orchids (Rzedowski, 2006; INEGI, 2009). TEF is located at elevations between 200 and 800 m, on slightly steep slopes and in small valleys (INEGI, 2009). Mean annual temperature is 24 °C, and precipitation ranges from 1,600 to 2,000 mm (Rzedowski, 2006; INEGI, 2009). Arboreal species are represented by the genera *Bursera*, *Ceiba*, *Cedrela*, *Lysiloma*, and *Piscidia*, which reach heights of 30–40 m (INEGI, 2009; Rzedowski, 2015). The agroecosystems SC and MH are located in sites that were previously MCF. The SC retains much of the arboreal stratum of MCF, and the arboreal species are from the genera *Citrus*, *Mangifera*, and *Pouteria*; there is selective pruning, and no phytosanitary control. MH is composed of corn, bean, chickpea, squash, chile pepper, tomato, citrus fruit, and guayaba crops, and occasionally isolated native trees of the genera *Quercus* and *Liquidambar*. GA is located in areas that were previously dominated by TEF on slightly steep slopes and valleys. The latter sites contain exotic grasses and occasionally isolated trees of the genera *Cedrela* and *Citrus* (INEGI, 2009; Rzedowski, 2015).

### Sampling design

We made 12 sampling field trips; six were conducted during the rainy season between June and November 2013, and six in the dry season between December 2013 and May 2014, covering the seasonal activity of amphibian species of the study area. Field trips had duration of 5 days each, assigning a day to each land-cover type (MCF, TEF, MH, SC, and GA). The sites were categorized according to the diagnostic characteristics of each land-cover type in the region: elevation, plant species, and terrain orography (see *study area*, section above), and with the help of biogeographic information from INEGI (2009) for the study area. For each land-cover type, six different sites were chosen with at least one km of separation between each one, in order to maintain the sampling sites as independent units (Cushman, 2006; Fig. 1). For each sampling sites, were made four transects (one per person) of 500 m long and variable in width, due to the marked heterogeneity of the terrain (i.e., badlands, steep slopes, rocky outcrops, vegetation physiognomy, etc.). All transects were oriented towards a cardinal point to avoid duplication (pseudo-replication) of the individual’s count (Badillo-Saldaña, Ramírez-Bautista & Wilson, 2016). The sampling effort was applied identically in each land-cover types, which consisted of four people sampling amphibians from 18:00 to 03:00 h in each one, which generated both 432 h and 24 km of sampling effort for each land-cover type.

The organisms were detected through a direct search method, reviewing the potential microhabitats from ground level up to three m in height, and considering the natural history of the study groups (Manzanilla & Péfaur, 2000; Amador, 2010). It is important to clarify that in MCF, TEF, and SC, there are probably species that live above three m, such as *Chiropterotriton* spp. or *Sarcohyla* spp., which were not detected by the type of sampling that was possible to carry out.

Specimens that could not be identified at the study sites were transported to the laboratory for later taxonomic determination. Collected specimens were deposited at the collection of amphibian and reptiles of the Centro de Investigaciones Biológicas of the Universidad Autónoma del Estado de Hidalgo. A collecting permit (SGPA/DGVS/02419/13) was issued to ARB by Secretaría de Medio Ambiente y Recursos Naturales (SEMARNAT) (2010). Scientific names were updated according to the most recent taxonomic changes (Rovito et al., 2015; Duellman, Marion & Hedges, 2016). Taxonomic verification of the species was made by Uriel Hernández-Salinas.

### Data analysis

For this study, three systems of conservation status were used, the Mexican standard NOM-059-SEMARNAT-2010, the IUCN Red List (IUCN, 2018), and the environmental vulnerability score (EVS) applied by Wilson, Johnson & Mata-Silva (2013), where a score is assigned to a native amphibian species based on its ecological distribution (types of vegetation inhabited by the species), geographical distribution, and reproductive mode (direct or indirect).

The sample coverage index was used to assess the completeness of the inventories. This index estimates the proportion of individuals of each species observed in a sample with respect to the total abundance (including observed and unobserved individuals) of the community, in this case, in each land-cover type (Chao & Tsung-Jen, 2003; López-Mejía et al., 2017). The sample coverage index standardizes communities sampled equally by extrapolation, to compensate for the dependence of the sample size and compares species richness when the number of individuals is not equal between communities (Moreno, 2001; Chao & Jost, 2012). Index values were determined with the following formula:

$${C_n} = {\rm{ }}1-{f_1}/n\left[ {\left({n-1} \right){f_1}/\left({n-1} \right){f_1} + 2{f_2}} \right],$$

where *f*_1_ is the number of species with a single individual in the sample, *f*_2_ is the number of species with two individuals in the sample, and *n* is the total number of individuals in the sample (Chao & Tsung-Jen, 2003). The resulting value of this index goes from 0 to 1, where 0 implies that none of the species in a land-cover type was found, and 1 that all the present species of a land-cover type have been detected.

The structure of amphibian communities was analyzed by rank-abundance curves for each land-cover type, using the relative abundance of each species (*p_i_*); which was graphed according to the natural logarithm of the proportion of each *p* (*n*/*N*) (where *N* is the total number of individuals present in the sample), ordering the data from the most abundant to the rarest species based on the specific richness (S; Magurran, 1988). Evenness between the land-cover types was estimated by Pielou’s evenness index (*J′*), using the formula:

$$J' = {{H'} \over {{{H'}_{{\rm{max}}}}}},$$

where *H′* is the Shannon–Wiener index and *H′*_max_ is equal to the natural logarithm of specific richness (ln S; Chao & Jost, 2012). The value of *J′* goes from 0 to 1, where 0 corresponds to situations where all species are not equally abundant and 1 when they are. Additionally, a detrended correspondence analysis (DCA) was done with the relative abundances of each of the six sites belonging to each land-cover type (MCF, TEF, SC, MH, and GA), to observe both the distribution of the species and the existence of species exclusive for each land-cover type (Waltert et al., 2005).

Land-cover type diversity was quantified using the diversity index (1*D*) proposed by Jost (2006), from which the number of effective species was obtained by the formula

$$1D = {\rm{ exp}}\left({H'} \right),$$

using the exponent *q* = 1; we chose this value because in this way all species receive a weight proportional to their abundance recorded in each land-cover type, that is, diversity index is obtained from the abundance of each of the species found, without favoring disproportionately common species (what would happen if a value of *q* > 1 were given) or rare species (if a value of *q* < 1 were given) (Jost, 2006). Beta diversity was evaluated by Jaccard’s dissimilarity index, whose values range from 0 to 1, where 0 means that land-cover types are identical, and 1 that the land-cover types are different (Legendre, 2014). Finally, total dissimilarity was divided into two components, replacement (β_3_) and differences in richness (β_rich_); beta analyses were performed with the vegan package (Oksanen et al., 2010), for the R ver. 3.3.1 program (Carvalho, Cardoso & Gomes, 2012).

The percentage of canopy cover of each site was estimated through the use of satellite images obtained from Google Earth Pro. The images corresponding to 2013 were used, and were georeferenced using the Georeferencing function of ArcGIS ver. 10.3 (Esri, Redlands, CA, USA). A buffer of one km in diameter was then established, using the beginning of sampling at each site as the starting point; the buffer was obtained through the Analysis Tools function of ArcGIS ver. 10.3 (Esri, Redlands, CA, USA). The polygons were superimposed on the satellite images, and a polygon was drawn following the perimeter of the canopy cover at each site using the GlobalMapper ver. 13 program. Subsequently, we generated a multiple linear analysis (MLA) for determine if there is a relationship between canopy cover and both amphibian richness and abundance in the study area. The MLA was carried out with the Past ver. 3.0 program (Hammer, Harper & Ryan, 2001), considering canopy cover as an independent variable, and both species richness and abundances as dependent variables.

## Results

A total of 595 individuals were recorded in this study. These individuals belong to 14 species, nine genera, six families and two orders (Table 1). Of these 14 species, six are under some risk category according to the Mexican standard NOM-059-SEMARNAT-2010, 13 by the IUCN, and seven have high vulnerability (EVS ≥14) based on Wilson, Johnson & Mata-Silva (2013; Table 1); in this sense, forest-depended species were recorded as *Aquiloeurycea cephalica*, *Craugastor decoratus*, and *C. rhodopis* (Ramírez-Bautista et al., 2014). Of the registered species, seven present direct reproductive mode and seven indirect (Table 1).

**Table 1:** Registered species in the study area.

Taxa	Land-cover types					Endemicity	Reproductive mode	NOM-059-2010	Red List IUCN	EVS
	MCF	TEF	SC	MH	GA					
AMPHIBIA										
CAUDATA										
Plethodontidae										
*Bolitoglossa platydactyla*		X				EM	ID	Pr	NT	15
*Aquiloeurycea cephalica*	X					EM	ID	A	NT	14
ANURA										
Bufonidae										
*Incilius nebulifer*	X	X	X	X	X	NEM	ID	Nc	LC	6
*Incilius valliceps*	X	X	X	X	X	NEM	ID	Nc	LC	6
*Rhinella horribilis*	X	X	X	X	X	NEM	ID	Nc	NC	3
Craugastoridae										
*Craugastor augusti*	X			X		EM	DD	Nc	LC	8
*Craugastor decoratus*	X					EM	DD	Pr	VU	15
*Craugastor rhodopis*	X					EM	DD	Nc	VU	14
Eleutherodactylidae										
*Eleutherodactylus longipes*	X		X	X		EM	DD	Nc	VU	15
*Eleutherodactylus verrucipes*	X	X	X	X		EM	DD	Pr	VU	16
Hylidae										
*Rheohyla miotympanum*	X	X	X		X	EM	ID	Nc	NT	9
*Smilisca baudinii*	X	X	X		X	NEM	ID	Nc	LC	3
Ranidae										
*Rana berlandieri*	X	X	X		X	NEM	ID	Pr	LC	7
*Rana johni*		X	X		X	EM	ID	P	EN	14
Total	12	9	9	6	7					

**Note:**

The letter X represent the presence of the species in each land cover types evaluated.

Land-cover types: MCF, mountain cloud forest; TEF, tropical evergreen forest; SC, shaded coffee; MH, milpa huasteca; GA, grazing area. Endemicity: EM, endemic to Mexico; NEM, no endemic to Mexico. Reproductive mode: direct development (DD) and indirect development (ID). Conservation status: NOM-059-SEMARNAT-2010; A, amenazada; Pr, sujetas a protección especial; P, en peligro de extinción; Nc, no considerada; IUCN Red List: NC, not considered; LC, least concern; NT, near threatened; VU, vulnerable; EN, endangered; EVS: Low vulnerability = 3–9; Medium vulnerability = 10–13; Hight vulnerability = 14–19.

**Figure 2:**
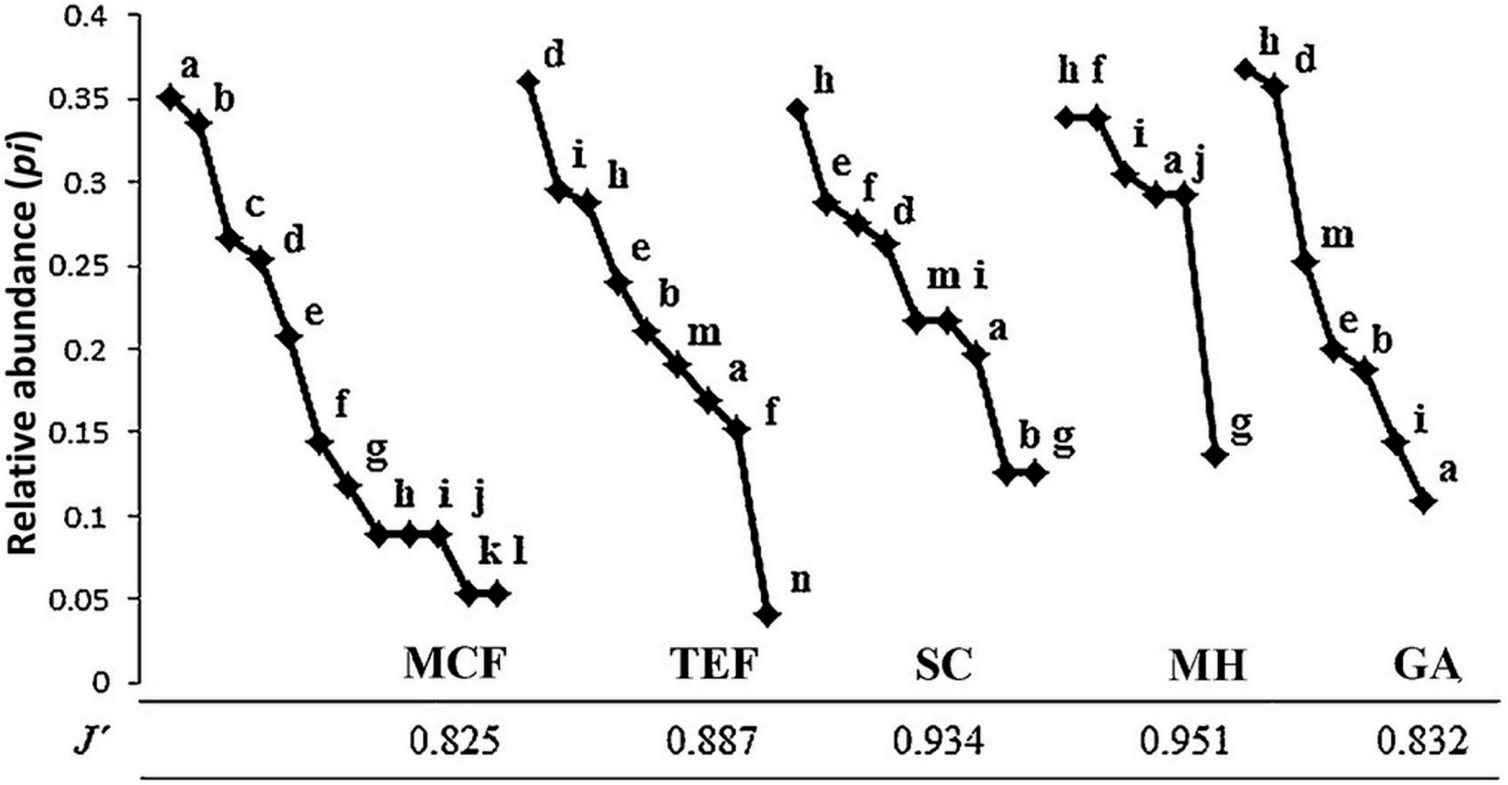
Abundance range curves. Land-cover types analyzed: mountain cloud forest (MCF), tropical evergreen forest (TEF), shade coffee (SC), milpa huasteca (MH), and grazing area (GA). Species: (a) *Incilius nebulifer*, (b) *Smilisca baudinii*, (c) *Craugastor rhodopis*, (d) *Rheohyla miotympanum*, (e) *Rana berlandieri*, (f) *Eleutherodactylus verrucipes*, (g) *E. longipes*, (h) *Rhinella horribilis*, (i) *I. valliceps*, (j) *Craugastor augusti*, (k) *C. decoratus*, (l) *Aquiloeurycea cephalica*, (m) *R. johni*, and (n) *Bolitoglossa platydactyla*.

Complete inventories (100%) were successfully obtained for all land-cover types, with the exception of the MCF (87%). MCF (58.3% ± 16% CC) showed the highest species richness with 12 species, followed by TEF (57% ± 13.5% CC) and SC (51.1% ± 5.5% CC) with nine species each. GA (18.5% ± 11.9% CC) and MH (32.5% ± 13.5% CC) showed the lowest richness, with seven and six species respectively (Table 1). *Incilius nebulifer* was the most abundant species in MCF (*n* = 22 individuals), while *Rheohyla miotympanum* was in the TEF (*n* = 69), and *Rhinella horribilis* was the most abundant species in anthropized environments (SC = 19, MH = 16, and GA = 47; Fig. 2). *Aquiloeurycea cephalica*, *C. decoratus*, and *C. rhodopis* were exclusive species to MCF, likewise, *A. cephalica* and *C. decoratus* were the least abundant species in MCF (Fig. 2); *Bolitoglossa platydactyla* was exclusive species and the least abundant to TEF (Fig. 2). *Smilisca baudinii* and *Eleutherodactylus longipes* were the least abundant species in SC, *E. longipes* in MH, and *I. nebulifer* in GA (Fig. 2). The most even land-cover type was MH (*J′* = 0.951), since the abundance of all recorded species was similar, while the MCF turned out to be the least even (*J′* = 0.825), having rare species and represented by few individuals (Table 1; Fig. 2).

**Figure 3:**
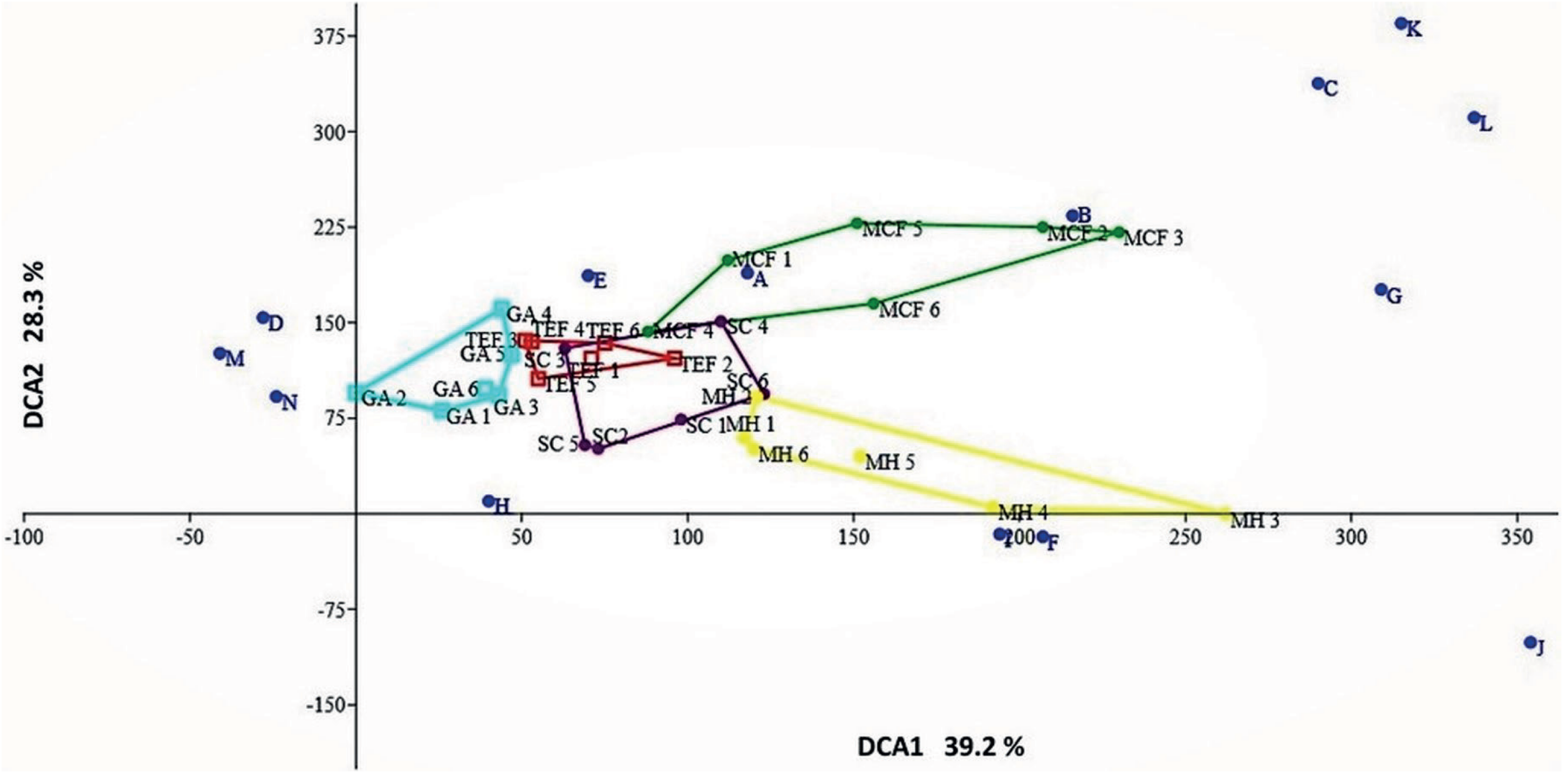
Detrended correspondence analysis. Ordination diagram of the function 1 and 2 from the DCA generated with the relative abundances of species recorded within each of sites belonging to each land-cover types: mountain cloud forest (MCF, green cluster); tropical evergreen forest (TEF, red cluster); shade coffee (SC, purple cluster); milpa huasteca (MH, yellow cluster); grassland area (GA, blue cluster). Species: (A) *Incilius nebulifer*, (B) *Smilisca baudinii*, (C) *Craugastor rhodopis*, (D) *Rheohyla miotympanum*, (E) *Rana berlandieri*, (F) *Eleutherodactylus verrucipes*, (G) *E. longipes*, (H) *Rhinella horribilis*, (I) *I. valliceps*, (J) *Craugastor augusti*, (K) *C. decoratus*, (L) *Aquiloeurycea cephalica*, (M) *R. johni*, and (N) *Bolitoglossa paltydactyla*.

Considering the relative abundances through the DCA, the most amphibian species have low preference for some type of environment; with the exception of species with direct reproductive mode (*A. cephalica*, *C. decoratus*, *C. rhodopis*), which inhabit only the MCF (Fig. 3); but also notes that other species of direct reproductive mode (*C. augusti*, *E. longipes*, and *E. verrucipes*) are highly related with the MH. On the other hand, it is observed that the SC presents a mixture of species that can be found in both TEF and MCF (Fig. 3).

**Figure 4:**
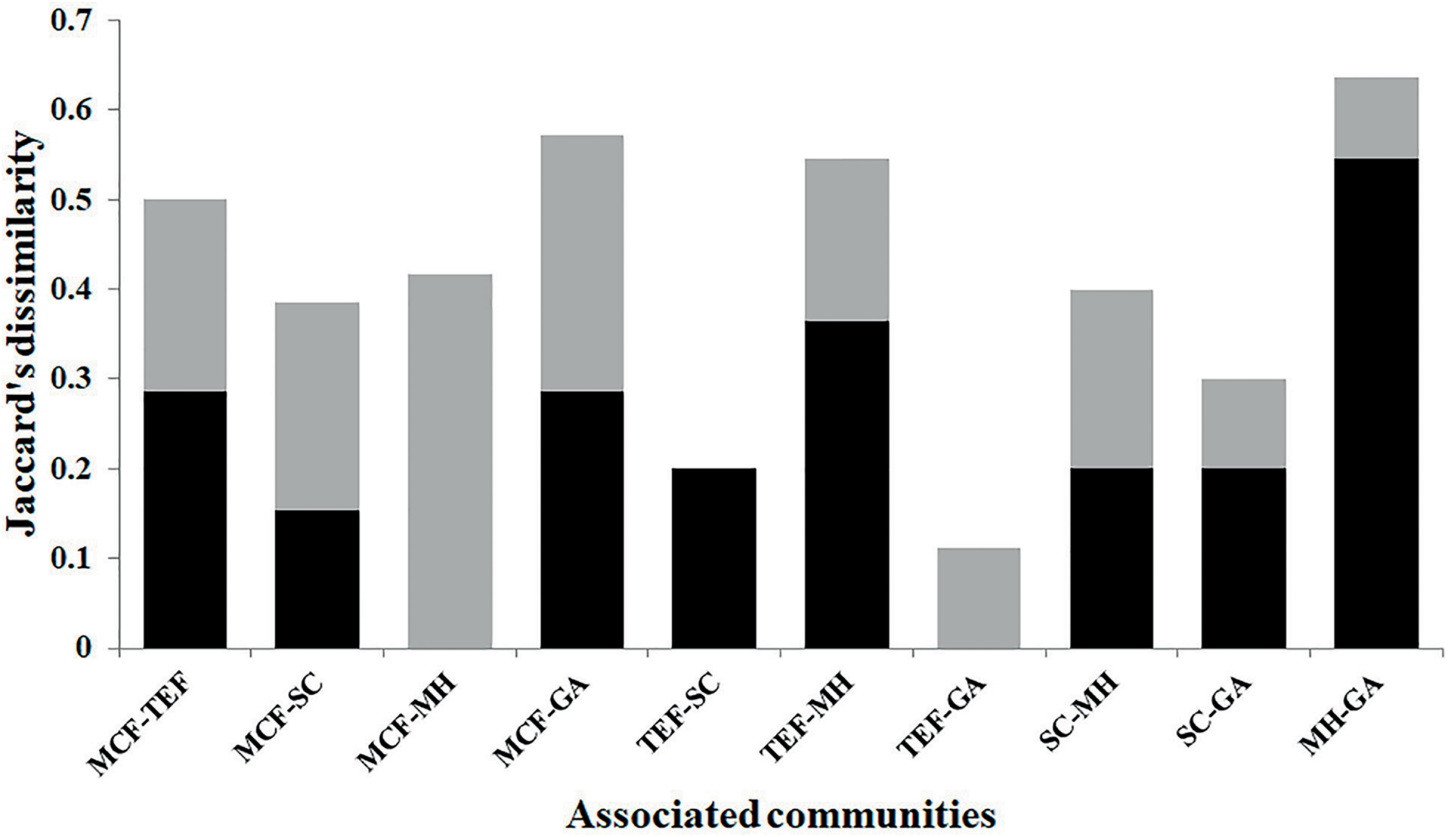
Beta diversity. Beta diversity by land-cover types: MCF, mountain cloud forest; TEF, tropical evergreen forest; SC, shade coffee; MH, milpa huasteca; GA, grazing area. The black color of the bars represent the replacement, and gray color show the difference in species richness.

According to the diversity index (1*D*), the SC showed the highest diversity, with 6.9 effective species, followed by the MCF with 6.1, TEF 5.9, MH 5.2, and lastly GA with 4.1. The Jaccard dissimilarity index indicated that the land-cover types analyzed showed high (0.63) and low (0.11) dissimilarity values (Fig. 4). The most different land-cover types were MH and GA, and these differences were due to species replacement; while the most similar were TEF and GA, due to a low difference in species richness (Fig. 4).

A regression analysis indicated that canopy cover did not influences both richness and abundance (*r*^2^ = 0.02, *p* = 0.03; Wilk’s Lambda = 0.78). Species richness decreased particularly with canopy cover loss (*r*^2^ = 0.45, *p* = 0.01), but the abundance was weakly related with canopy cover loss (*r*^2^ = 0.15, *p* = 0.4).

## Discussion

The greatest species richness was found in the environments containing canopy cover (MCF, TEF, and SC), which highlights the importance of the condition generated by the arboreal stratum for amphibian species (Cushman, 2006; Santos-Barrera & Urbina-Cardona, 2011). Consequently, the evident reduction of canopy cover in MH, and the almost complete loss in GA, suggests adverse conditions for this group, which needs a moist environment to hydrate and carry out adequate gas exchange, as well as bodies of water to reproduce (Young et al., 2004; Trimble & Van Aarde, 2014).

Although the highest species richness values were observed in primary forest communities, the greatest evenness was found in the traditional agroecosystems (SC and MH) because these land-cover types showed similar abundances in all species recorded in these environments. In this sense, the increase in the uniformity of species could be an indicator of disturbance in a community, since when forests are disturbed, especially tropical ones; the first species that disappear are those that are considered rare due to their specific ecological requirements. Additionally, amphibian communities recorded in agroecosystems are represented by species related to undisturbed forest (e.g., *C. augusti* and *E. longipes*) and sites with a lack of canopy cover (e.g., *I. valliceps*, *R. horribilis*, and *R. berlandieri*); probably these environments have bioclimatic characteristics (temperature and humidity) intermediate between primary forests and clearly disturbed sites, such as GA (Trimble & Van Aarde, 2014; Cruz-Elizalde et al., 2016), or it might be because there are fewer species to compete for available resources. Although SC and MH showed similar evenness values, the SC had a larger number of species, including all the species that were recorded in MH; therefore, the SC plays a more important role at the landscape level in the conservation of amphibians, not so the MH.

Shade coffee showed the greatest diversity even though it had lower richness than the MCF; however, it should be taken into account that inventory completeness in the SC where 100%, unlike the MCF where a completeness of 87% was reached. The SC has larger species evenness, so the effective number of species was greater. This result differs from other studies conducted in Mexico, which reported a greater diversity of amphibians in MCF than in SC (Pineda & Halffter, 2004; Murrieta-Galindo et al., 2013). This is likely due to considerable variation in amphibian assemblages and bioclimatic characteristics of MCF throughout their distribution (Gual-Díaz & Goyenechea, 2014; Ruiz-Jiménez, Téllez-Valdés & Luna-Vega, 2012); therefore, each assembly could be responding differently to habitat modifications. However, the present study and those carried out by Pineda & Halffter (2004) and Murrieta-Galindo et al. (2013) find that although the SC presents richness similar to the MCF, the assemblages of species are different in each land-cover type, and that the primary forests are the ones that present exclusive species. Additionally, coffee plantations are managed in diverse ways; therefore, some that have different bioclimatic characteristics to the primary forests, such as monoculture sun coffee plantations, could be unfavorable for the conservation of amphibian diversity (Hernández-Martínez, 2008). It is therefore necessary to conduct studies that consider the effect of agroecosystems in terms of their characteristics, such as size, the number of associated tree species, the degree of management (pruning and use of agrochemicals), and elevation, among others (Gallego-Ropero, 2005). This could suggest good agricultural management practices that increase productivity and reduce negative effects on biodiversity.

The slight difference in species composition between the land-cover types may be due to a nesting effect; however, most of the land-cover types comparisons showed higher values for replacement (51% ± 32%) than differences in species richness (48% ± 32%; Carvalho, Cardoso & Gomes, 2012). These results do not show any trend. On the other hand, the majority of the species that make up the regional assemblage inhabit both native and non-native land-cover types, because they have physiological characteristics that enable them to partially resist the microclimatic changes produced by the establishment of agricultural systems (Trimble & Van Aarde, 2014). The proximity of native forests to the agroecosystems causes a lax transition between these environments, especially between shade-grown coffee and undisturbed forests (Macip-Ríos & Muñoz-Alonso, 2008), which could explain the low beta diversity observed in the SC (Fig. 3).

The moderate dissimilarity (*J* = 0.63) found between MH and GA could be due to species with the ability to reproduce in drier habitats. In these environments, there is an 86% change in species composition due to the replacement of species with a direct reproductive mode (*E. longipes, E. verrucipes*, and *C. augusti*) by species with an affinity to drier sites (*R. miotympanum*, *R. berlandieri*, and *R. johni*). It is necessary to emphasize that MH areas were established in sites that were previously MCF, while the GA areas were TEF; therefore, the changes in species composition between MH and GA could be a product of the reduction in environmental humidity, since the tropical forests of the north of the Sierra Madre Oriental have a greater degree of evaporation and higher temperatures than the open sites of MCF (Badillo-Saldaña, Ramírez-Bautista & Wilson, 2016). Variation in relative humidity is likely due to MCF areas having greater precipitation and constant foggy conditions throughout the year (INEGI, 2009; Ruiz-Jiménez, Téllez-Valdés & Luna-Vega, 2012), allowing species with direct development to remain in sites devoid of litter or canopy cover, such as MH. Additionally, a large number of rocks and crevices in this environment may be functioning as suitable microhabitats for anurans with direct development, such as species of the genera *Craugastor* and *Eleutherodactylus*. In contrast, GA has higher ambient temperatures and lower humidity since it is located in areas previously dominated by TEF and lacking canopy cover; therefore, it is mostly generalist species that are able to occupy this kind of environment. This is the first study of amphibians carried out in the study area and aimed at determining the effect of environments modified by an anthropic effect. This study provides a reference framework for estimating the effect on amphibians of anthropogenic modifications within the types of agroecosystems in the transition zones of two biogeographic provinces in the central region of Mexico.

In summary, the effect of anthropic modification of MCF and TEF is clearly seen in the reduction of amphibian diversity, which is closely related to the loss of canopy cover (Blaustein & Kiesecker, 2002; Trimble & Van Aarde, 2014), and supports the theory that primary forests are irreplaceable to conserve tropical biodiversity, both in amphibians and in other groups of vertebrates, insects and plants (Gibson et al., 2011). Therefore, to preserve the amphibian diversity of the study area, it is necessary to ensure the presence of the last fragments of original forest that still exist, primarily the remaining MCF. In addition to containing the greatest species richness, it is the only environment that harbors exclusive species, as well as the most vulnerable according to the conservation status assigned by SEMARNAT, IUCN, and the EVS system. Additionally, supporting the maintenance of current areas with traditional agricultural systems (as opposed to monocultures) such as SC will help secure high diversity and species evenness similar to those found in primary forest. Finally, it is recommended that implementation of conservation policies also take into account reduction and optimization of GA, since the latter were shown to significantly affect amphibian species evenness and diversity.

## Supplemental Information

10.7717/peerj.6390/supp-1 Supplemental Information 1 Database: raw data of the richness and abundance and the species found in the study. The richness and abundance of the species found in four types of vegetation on 12 field trips.

## Additional Information and Declarations

### Field Study Permissions

The following information was supplied relating to field study approvals (i.e., approving body and any reference numbers): Colleting permit (SGPA/DGVS/02419/13) was issued by SEMARNAT (Secretaria del Medio Ambiente y Recursos Naturales). We only gathered observational records.

### Data Availability

The following information was supplied regarding data availability: The raw data are available in a Supplemental File.
